# 159. Characterization of Suboptimal Discharge Antimicrobial Prescriptions and Effect of Inpatient Audit and Feedback on Quality of Antimicrobial Prescribing

**DOI:** 10.1093/ofid/ofab466.361

**Published:** 2021-12-04

**Authors:** Lauren M Puckett, Laura Bio, Sean Cornell, Torsten Joerger, Hayden T Schwenk, Hayden T Schwenk

**Affiliations:** 1 Lucile Packard Children’s Hospital Stanford, Stanford, California; 2 Stanford Children’s Health, Palo Alto, CA; 3 Stanford University School of Medicine, Stanford, California; 4 Stanford University, Stanford, CA

## Abstract

**Background:**

Approximately 30% of children are discharged from the hospital with an antimicrobial prescription; nearly a third of these prescriptions are suboptimal. Although the best approach to antimicrobial stewardship of discharge prescriptions remains uncertain, prospective audit and feedback (PAF) has improved inpatient antimicrobial use. We aimed to identify and characterize suboptimal discharge antimicrobial prescribing and assess the impact of inpatient PAF on the quality of discharge antimicrobial prescribing at a free-standing children’s hospital.

**Methods:**

A retrospective review of enteral discharge antimicrobial prescriptions between 12/1/20-5/31/21 and parenteral antimicrobial prescriptions sent to our hospital’s infusion pharmacy between 3/1/21-5/31/21 was performed to determine if suboptimal or not. A prescription was determined to be suboptimal if the antimicrobial choice, dose, frequency, duration, formulation, or indication was not consistent with institutional and/or national guidelines. Data collection included the antimicrobial, indication, and prescribing medical service. Prescriptions were evaluated for a corresponding inpatient PAF for the same drug and indication and then stratified based on inpatient PAF completion.

**Results:**

A total of 1192 discharge prescriptions for 698 unique patients over 834 hospital encounters were reviewed. Overall, 243 (20%) prescriptions were identified as suboptimal; reasons were duration (16%), dose (8%), frequency (5%), or antimicrobial choice, formulation, or route (≤1%). Prescriptions for cephalexin had the highest rate of suboptimal prescribing (80/167, 48%), followed by amoxicillin-clavulanate (89/203, 44%). A corresponding inpatient PAF was identified for 675 (57%) of discharge antimicrobial prescriptions. Inpatient PAF prior to discharge resulted in fewer suboptimal discharge prescriptions for the same antimicrobial (8% vs. 36%, p < 0.001).

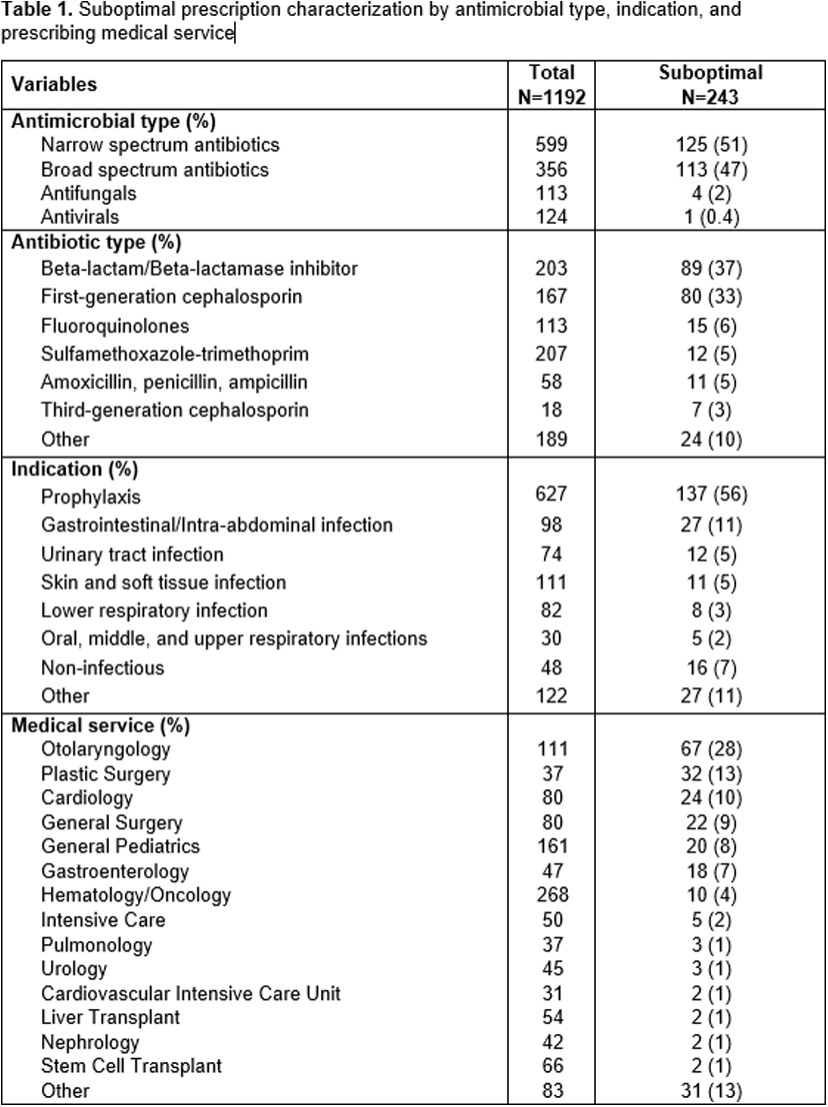

**Conclusion:**

Antimicrobial prescribing at inpatient discharge was suboptimal in 1 of every 5 prescriptions. Inpatient PAF was associated with improved antimicrobial prescribing at hospital discharge. Antimicrobial stewardship programs should continue to explore ways to capture and intervene on antimicrobials prescribed at discharge.

**Disclosures:**

**Hayden T. Schwenk, MD, MPH**, Nothing to disclose

